# Giant pedunculated cellular angiofibroma of the labia majora: a case report

**DOI:** 10.1186/s13256-025-05803-0

**Published:** 2026-01-21

**Authors:** Mudathir Bafadni, Khalid Abu Aagla

**Affiliations:** 1 General Surgery Department, Faculty of Medicine, Alazhari University, Khartoum North, Sudan; 2 General Surgery Department, Faculty of Medicine, National Ribat University, Khartoum, Sudan; 3 General Surgery Department, Prince Abdul Mohsen General Hospital, AlUla, Saudi Arabia

**Keywords:** Cellular angiofibroma, Vulva, Genital mesenchymal tumors

## Abstract

**Background:**

Cellular angiofibroma is a rare, benign mesenchymal tumor most commonly arising in the vulvovaginal region in middle-aged to elderly women. Despite its indolent nature, it is frequently misdiagnosed due to a nonspecific clinical presentation and its resemblance to other vulvar masses.

**Case report:**

We present a case of a 65-year-old postmenopausal woman with a neglected, slowly growing giant pedunculated mass on her left labia majora that developed over 5 years. The patient underwent surgical excision and cosmetic reconstruction, with an uneventful postoperative recovery. Histopathological examination confirmed a benign myxoid spindle cell neoplasm consistent with cellular angiofibroma.

**Discussion and conclusion:**

This case highlights the challenges in the diagnosis of cellular angiofibroma owing to the tumor’s often nonspecific presentation and emphasizes the importance of surgical excision as the standard treatment, despite a low reported risk of recurrence even with positive surgical margins.

## Introduction

Cellular angiofibroma, first described in 1997, is an uncommon benign tumor of the connective tissue[1, 2]. It primarily affects the genitourinary region in both sexes, with a predilection for the vulvar region in middle-aged females and the inguinoscrotal area in men[1, 2]. Histologically, it is characterized by a biphasic composition of bland, plump spindle cells and numerous prominent, small- to medium-sized, thick-walled hyalinized blood vessels set in a variably edematous or collagenous stroma[3]. These features are crucial for differentiating it from mimics such as angiomyofibroblastoma, which has more prominent epithelioid cells, and aggressive angiomyxoma, which is typically more infiltrative and hypocellular. Complete local surgical excision is the definitive standard of care, as nonsurgical treatments such as radiation or chemotherapy are not indicated for this benign condition[4]. The prognosis is excellent, with no reported cases of metastasis and a very low risk of local recurrence, which occurs in less than 5% of cases, even with positive surgical margins[5, 6]. Herein, we report a case of a giant, neglected cellular angiofibroma of the labia majora, noteworthy for its exceptional size and the significant delay in presentation, which highlights the clinical and sociocultural challenges associated with this rare tumor.

## Case report

A 65-year-old postmenopausal woman presented with a slowly growing mass on her left labia that had been present for 5 years. The mass originated 5 years prior as a small, asymptomatic lesion on the inferior part of the left labia and had progressively enlarged since. The patient delayed seeking medical attention due to sociocultural barriers and apprehension regarding potential surgical intervention.

More recently, the significant growth of the mass led to constant dragging pain, ambulation difficulty, and surface ulceration, which created daily hygiene challenges due to serous discharge.

**Fig. 1:**
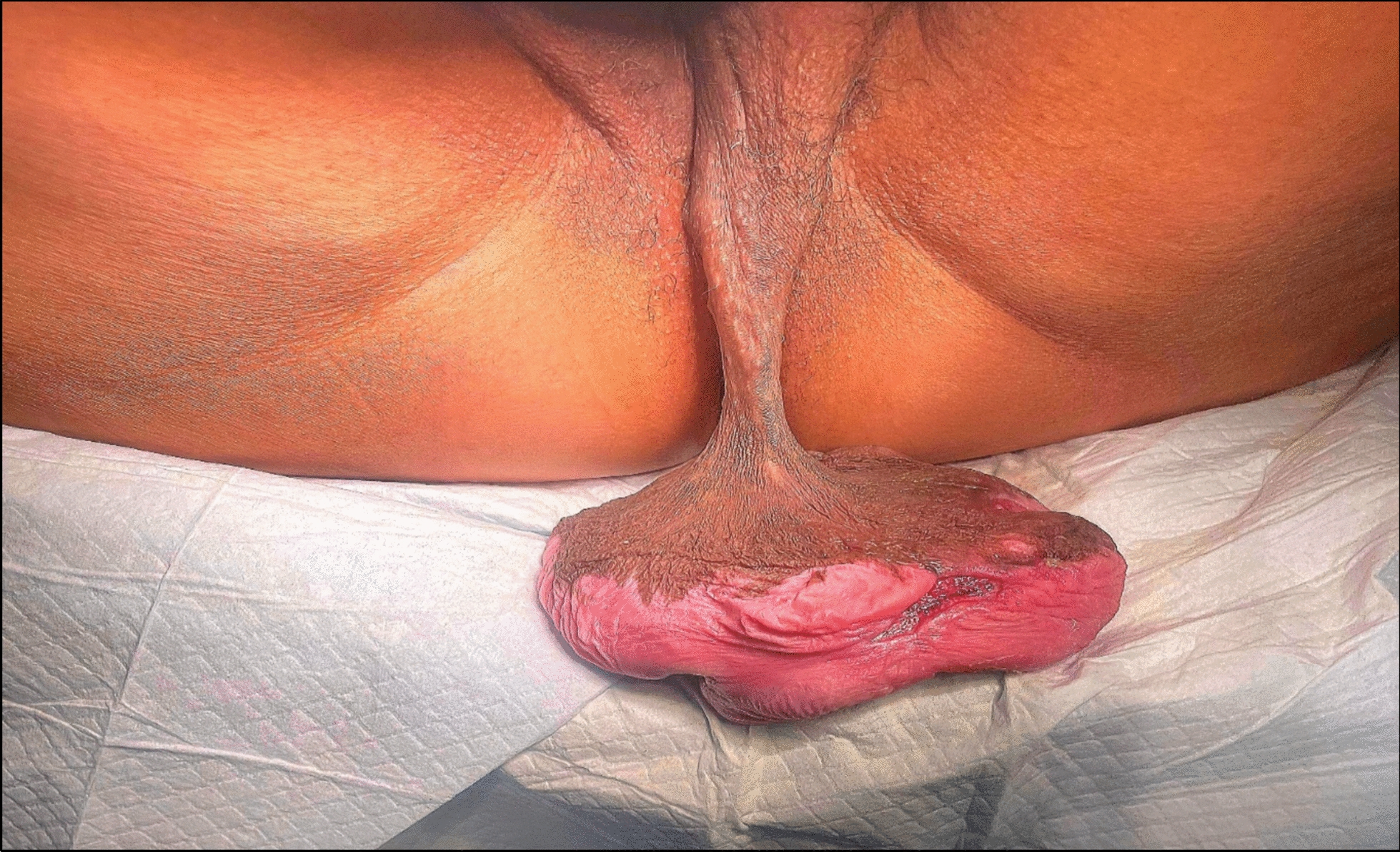
Giant pedunculated mass of the left labia majora

Upon examination, a large, pedunculated mass was observed arising from the lower edge of the left labia majora. The mass was attached by a 10 cm stalk. Its inferior surface showed areas of ulceration with associated serous discharge (Fig. 1).

**Fig. 2:**
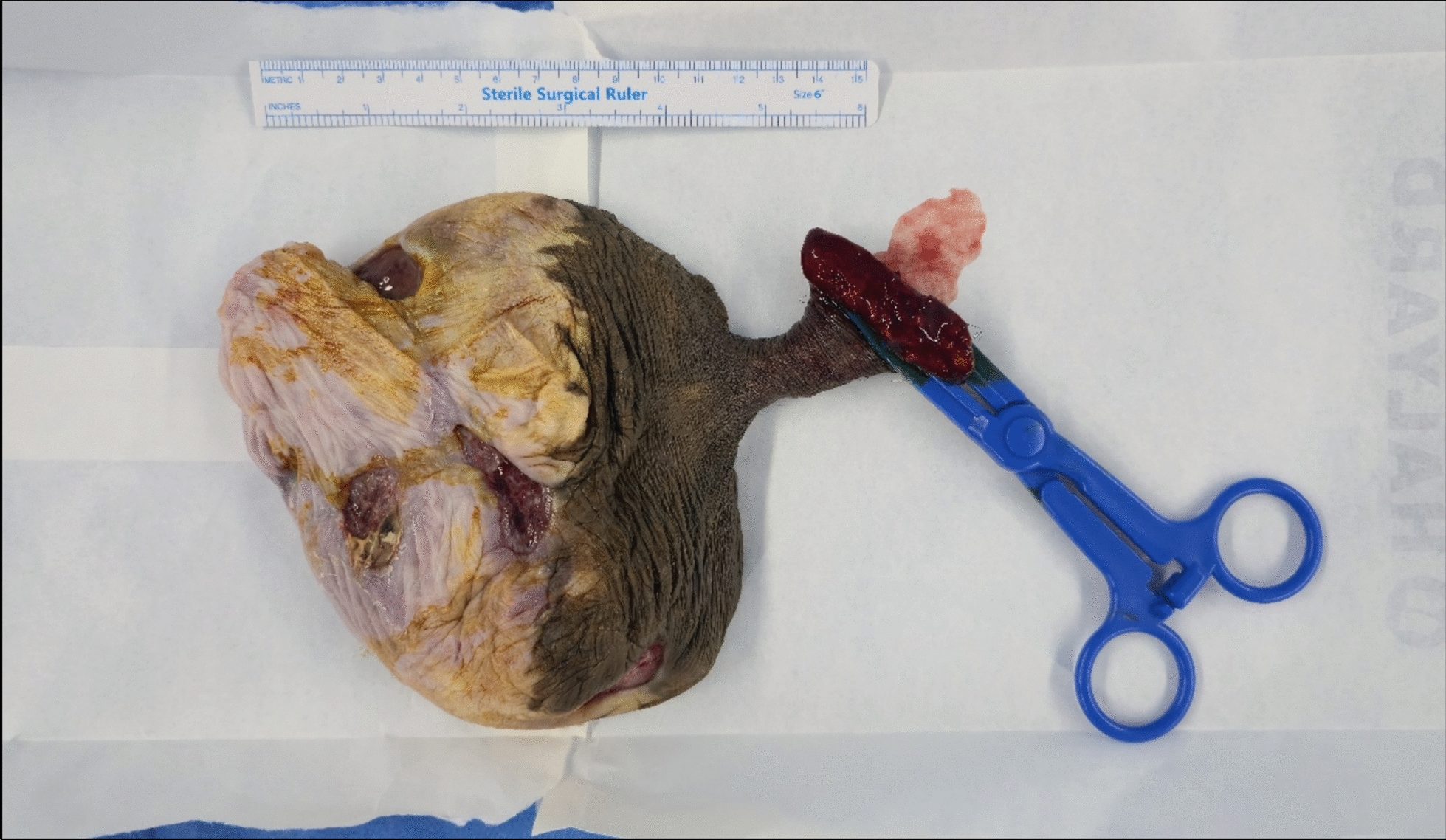
Resected specimen with the ulcerating inferior surface

The patient underwent surgical excision of the mass under spinal anesthesia. A longitudinal elliptical incision was made at the base of the stalk. A distinct feeding vessel was identified and ligated, and the mass was excised completely. The procedure was concluded with a cosmetic reconstruction of the left labia. The resected mass measured 12 × 11 × 8 cm and weighed 560 g (Fig. 2), which is significantly larger than the average reported size of approximately 3.6 cm for vulvar cellular angiofibromas.

**Fig. 3:**
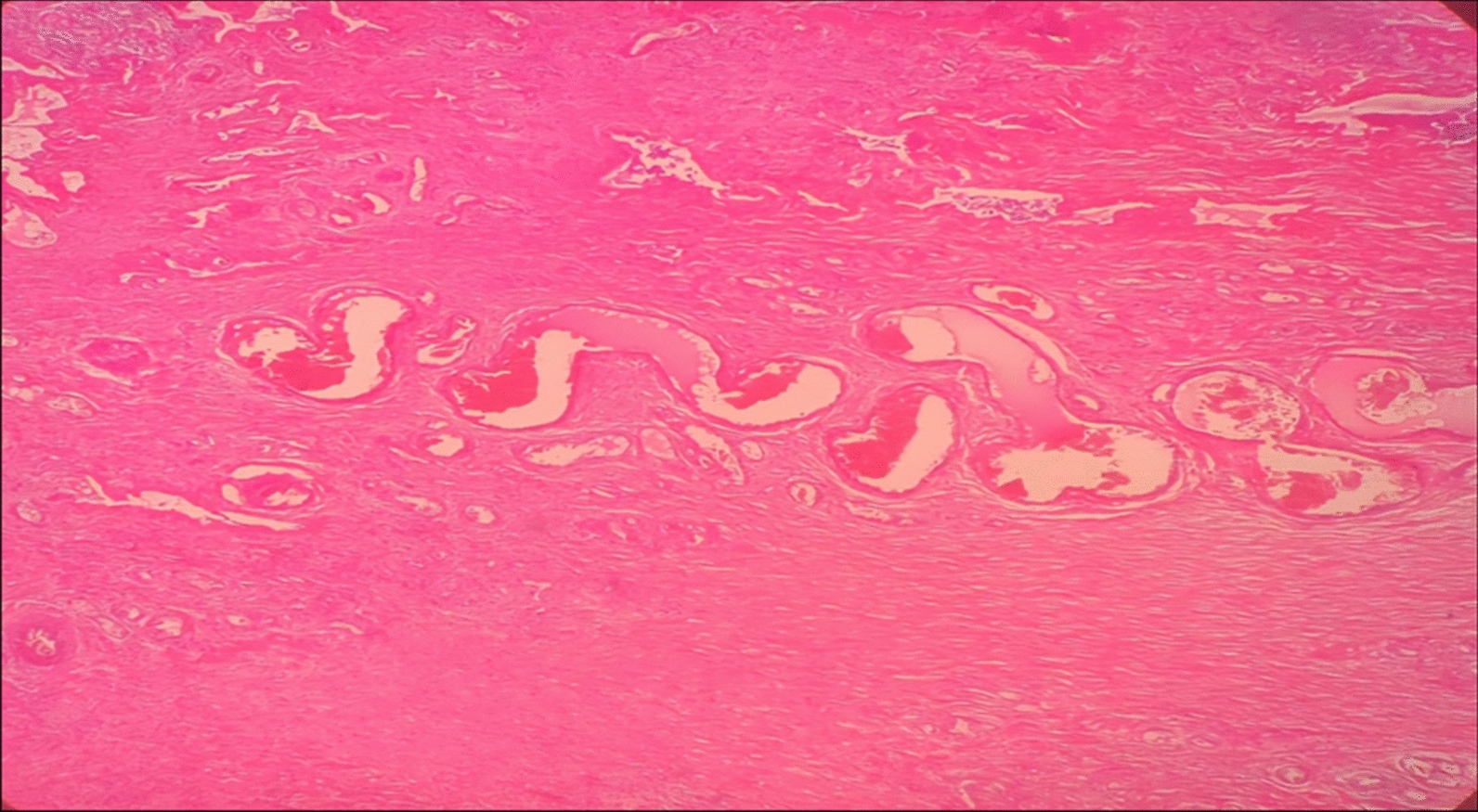
Section shows a spindle cell neoplasm composed of benign spindle cells with an admixed population of thick, hyalinized medium-sized blood vessels. No significant cytological atypia, mitotic activity, or necrosis was identified

The postoperative course was uneventful, and the wound healed well. Histopathological examination revealed a benign myxoid spindle cell neoplasm. Microscopically, the tumor was composed of bland, uniform spindle cells admixed with numerous thick-walled, hyalinized medium-sized blood vessels (Fig. 3). No significant cytological atypia, mitotic activity, or necrosis was identified, confirming the diagnosis of cellular angiofibroma. While immunohistochemistry was not performed, these tumors typically show positivity for vimentin and CD34, and are negative for desmin and S-100 protein (Fig. 3).

## Discussion

Cellular angiofibroma typically presents as a painless, well-defined lump, most often in the labia majora or perineum. While sizes average 3.6 cm, they can range from less than 1 cm to over 20 cm, with cases similar to ours being considered “giant.” Upon physical examination, vulvar angiofibroma typically presents as a well-circumscribed, solid, soft, mobile, or rubbery mass, which is grayish-white and can have a polyp-like or multi-lobed appearance [7, 8]. Notably, these masses typically lack signs of infection, inflammation, or lymphadenopathy. These nonspecific characteristics frequently lead to clinical misdiagnosis, with the mass often mistaken for other benign vulvar conditions such as a Bartholin’s gland cyst, vulval cyst, leiomyoma, lipoma, or even an inguinal hernia. The presentation in our case, as a long-stalked giant polyp, is consistent with the varied morphology reported in the literature [6, 7]. Although it predominantly arises in the inguinoscrotal area in men, both extragenital and, less frequently, extrapelvic manifestations have been observed and reported in the medical literature [8].

The precise etiology and pathogenesis of cellular angiofibroma remain poorly understood. Several theories have been proposed. The most supported are the angiogenic and histogenetic hypotheses, which point to an underlying vascular or connective tissue origin. Other potential etiologic factors under investigation include the influence of sex hormones, given its predilection for genital areas, and specific genetic alterations[4].

Accurately diagnosing vulvar angiofibroma depends largely on histopathological examination combined with immunohistochemistry. This is because the tumor’s clinical presentation and imaging results are often nonspecific, frequently leading to misdiagnosis [2, 9]. Microscopically, CA is characterized by its distinct biphasic pattern: a cellular component of bland, uniform spindle-shaped cells and a prominent vascular component of numerous thick-walled, hyalinized vessels [7, 10]. The stromal background can vary from edematous to collagenous. Immunohistochemically, the spindle cells are typically positive for CD34 and vimentin, which aids in diagnosis. While definitive diagnosis requires tissue sampling, preoperative imaging can be useful. Ultrasonography typically shows a well-defined, solid, hypoechoic mass with variable vascularity. On magnetic resonance imaging (MRI), cellular angiofibroma often appears as a well-circumscribed mass with intermediate signal intensity on T1-weighted images and heterogeneous high signal intensity on T2-weighted images, reflecting its mixed cellular, edematous, and vascular composition [7, 11]. These features can help differentiate it from purely cystic lesions, such as a Bartholin’s gland cyst or fatty tumors such as a lipoma.

The treatment of choice is complete local surgical excision to achieve clear margins. The tumor follows a benign course with excellent prognosis and no metastatic potential [12–14]. Interestingly, recurrence is uncommon even when surgical margins are positive, with one study noting a need for re-excision in only 27.8% of such cases [8]. This low recurrence risk suggests that overly aggressive resections may not always be necessary, particularly in sensitive anatomical locations.

Delayed presentation, as seen in our case, is common for cellular angiofibroma. This is primarily because the tumor is typically asymptomatic and slow-growing in its early stages, often going unnoticed until it becomes large enough to cause discomfort or functional issues [6, 7, 14, 15]. Compounding this delay, the nonspecific clinical presentation often leads to misdiagnosis as more common vulvar conditions, and the limitations of preoperative imaging can further postpone accurate identification [9, 12]. In our case, a significant factor contributing to the delayed presentation was the sociocultural stigma associated with genital health issues, which can be a powerful barrier to seeking timely medical care, particularly in some cultural contexts. Studies have shown that patient embarrassment and reluctance to undergo examination for gynecological conditions can lead to significant delays in diagnosis and treatment across various populations [16, 17]. This cultural barrier often results in patients postponing care until symptoms become severe or functionally limiting, as demonstrated in this case**.**

## Conclusion

Angiofibroma of the labia majora is a rare benign mesenchymal tumor. The nonspecific clinical features of this neoplasm can lead to misdiagnosis, emphasizing the importance of histopathological confirmation. There is no risk of recurrence with complete surgical excision, which provides an excellent prognosis even when there are positive surgical margins. There is particular significance to this case because of the tumor’s significant size and the 5-year delay in presentation caused by sociocultural barriers. Cellular angiofibroma should be considered when diagnosing vulvar masses and patient-specific factors may delay treatment.

## Data Availability

Not applicable.
